# Editorial: Advanced manufacturing of alternative microalgal-based bioproducts

**DOI:** 10.3389/fbioe.2023.1286213

**Published:** 2023-10-17

**Authors:** Wangbiao (Seven) Guo

**Affiliations:** Microbial Sciences Institute, Yale University, New Haven, CT, United States

**Keywords:** microalga biomass, CCUS, DHA, bioproduct, CO2 fixation

As the world grapples with the urgent Research Topic of climate change, the need for decarbonization solutions is escalating across various sectors, such as agriculture, maritime transport, and the manufacturing of iron, steel, and chemicals. Carbon capture, utilization, and storage (CCUS) technologies stand out as particularly promising, offering the potential to slash industrial carbon emissions by more than half within the next few decades. One innovative CCUS approach uses oxygenic microorganisms in photosynthetic cellular factories to transform flue gas CO_2_ into valuable biochemicals such as alternative feedstocks. However, the end products of this technology are still in the developmental stage, thus necessitating in-depth research that encompasses food production, feedstocks, cosmetics, and essential fatty acids. The journey toward the commercialization and broad-scale deployment of microalgae-based solutions is fraught with challenges, primarily technical and economic in nature. These challenges include the need to minimize the substantial energy requirements for harvesting, mitigate environmental risks, and facilitate access to high-value products.

Our objective is to accelerate advancements in the industrialization of microalgae technologies, spanning from micro-genetic engineering and metabolic pathway studies to the integration of new materials and processes, ultimately aiming for large-scale production. We aspire to the positive impact of the cost-effective and energy-efficient production of microalgae on both public health and global economic stability. To this end, we have curated this Research Topic titled “Advanced Manufacturing of Alternative Microalgal-Based Bioproducts.” We invited researchers to share their groundbreaking research exploring innovative methods for large-scale microalgal production using waste nutrients as alternative raw materials and in-depth studies on the unique functional characteristics of microalgal components to further their practical applications. After approximately a year of concerted effort, we have successfully compiled four pioneering research articles. As this research initiative concludes, it is crucial to acknowledge the transformative contributions these works have made to the field of Bioprocess Engineering. The articles in this Research Topic not only tackle urgent challenges related to climate change and sustainability but also introduce cutting-edge solutions via microalgal biotechnology.

The study conducted by Zhang et al. investigates how various bicarbonates impact the CO_2_ absorption capabilities of Spirulina in a carbon dioxide absorption hybrid with microalgae conversion (CAMC) system. Given the specific nature of emissions from power plants and the need to lower the costs of CO_2_ capture, the CAMC system offers a solution that sidesteps Research Topic such as heat loss during absorbent regeneration and nutrient depletion during microalgae cultivation. In this research, bicarbonate solutions derived from Na_2_CO_3_ and K_2_CO_3_ serve as the carbon sources for the growth of genetically modified Spirulina platensis. The study experimented with different bicarbonate concentrations to identify the most effective carbon source. The findings revealed that NaHCO3 outperformed K_2_CO_3_ as a medium in the CAMC system, promoting better growth of the modified Spirulina. This research offers a viable pathway for power systems to move toward carbon neutrality and is pivotal for the advancement of effective carbon capture solutions involving microalgae. The article was chosen for its immediate relevance to climate change mitigation through groundbreaking biotechnological approaches.

The study (Zhang et al.) focusing on the biosynthesis of Docosahexaenoic acid (DHA)-phospholipids via lipase-catalyzed transesterification stands out as a milestone in the field of green chemistry. This research not only introduces an eco-friendly way to produce omega-3-enriched substances but also investigates the underlying catalytic processes. The use of DHA-rich phosphatidylcholine (PC) has garnered considerable interest for its health-promoting properties in both the food and pharmaceutical sectors. In this particular study, untreated edible algal oil, abundant in DHA-triacylglycerol (DHA-TAG), was employed for the first time as a DHA source in the transesterification of phospholipids. This led to the creation of three unique types of phospholipids: DHA-PC, DHA-phosphatidylethanolamine (DHA-PE), and DHA-phosphatidylserine (DHA-PS). Various forms of DHA donors, such as fatty acids, methyl esters, and triglycerides, were also compared. Molecular dynamics (MDs) simulations were employed to elucidate the catalytic mechanisms at a molecular level, including substrate diffusion, the structure–activity relationship, and the influence of water content. This article was selected for its groundbreaking contributions to sustainable chemistry and its promising applications in the field of nutraceuticals.

The research conducted by Peng et al. presents an innovative technique for extracting polysaccharides from Chlorella species using microwave-assisted enzymatic extraction (MAEE). The conditions for this extraction method were fine-tuned using the Box–Behnken design and response surface methodology. The MAEE technique employed in this study demonstrated a high yield for CSCP extraction, offering an efficient approach for obtaining polysaccharides from Chlorella species. This pioneering research not only optimized the extraction process but also enhanced the yield and antioxidant properties of the polysaccharides. These advancements hold significant potential for applications in the food and pharmaceutical sectors, where there is a growing demand for compounds rich in antioxidants.

In the final article, Bi et al. explore the complex biosynthetic pathways involved in the production of sterols and fatty acids in the oil-rich marine microorganism *Schizochytrium* sp. Sterols are essential for both the structural and functional aspects of eukaryotic cells. In *Schizochytrium* sp. S31, the primary sterols produced are cholesterol, stigmasterol, lanosterol, and cycloartenol. However, the specific pathways and functional roles of sterol biosynthesis in this organism have not been fully identified.

Utilizing genomic data analysis and chemical biology techniques, the researchers were the first to digitally map out the mevalonate and sterol biosynthesis pathways in *Schizochytrium*. The findings suggest that due to the absence of plastids, *Schizochytrium* likely uses the mevalonate pathway, similar to fungi and animals, to produce isopentenyl diphosphate, a precursor for sterol synthesis. Additionally, the study uncovered the hybrid nature of the *Schizochytrium* sterol biosynthesis pathway, displaying characteristics of both algal and animal pathways.

Overall, this article lays a foundational understanding of the biosynthetic pathways in *Schizochytrium*, particularly focusing on the co-regulation mechanisms between sterol and fatty acid synthesis. This knowledge is pivotal for future engineering efforts aimed at sustainable lipid and high-value chemical production. The article was selected for its groundbreaking potential to transform the biofuel sector.

The choice to include these articles in this Research Topic was guided by their excellent technical and scientific quality and their collective contribution to the broader objective: the advancement of microalgal bioprocess engineering for a more sustainable future. Each article brings a unique perspective and solution to this goal, whether through innovative extraction methods, detailed exploration of biosynthetic pathways, sustainable chemistry techniques, or mechanisms for carbon capture. This research Research Topic stands out as a powerful endorsement of the transformative capabilities of microalgal biotechnology in tackling some of today’s most urgent environmental and sustainability challenges. We anticipate that these contributions will catalyze additional research and technological breakthroughs in the field, propelling us toward a more sustainable and environmentally responsible future.

